# Microstructure of the Surface of the Tongue and Histochemical Study of the Lingual Glands of the Lowland Tapir (*Tapirus terrestris* Linnaeus, 1758) (Perissodactyla: Tapiridae)

**DOI:** 10.3390/ani10122297

**Published:** 2020-12-04

**Authors:** Karolina Goździewska-Harłajczuk, Pavla Hamouzová, Joanna Klećkowska-Nawrot, Karolina Barszcz, Petr Čížek

**Affiliations:** 1 Department of Biostructure and Animal Physiology, Faculty of Veterinary Medicine, Wrocław University of Environmental and Life Sciences, 51-361 Wrocław, Poland; joanna.kleckowska-nawrot@upwr.edu.pl; 2 Department of Physiology, Faculty of Veterinary Medicine, University of Veterinary and Pharmaceutical Sciences Brno, 61242 Brno, Czech Republic; hamouzovap@vfu.cz; 3 Department of Morphological Sciences, Faculty of Veterinary Medicine, Warsaw University of Life Sciences, 02-776 Warsaw, Poland; karolina_barszcz@sggw.pl; 4 Department of Anatomy, Histology and Embryology, Faculty of Veterinary Medicine, University of Veterinary and Pharmaceutical Sciences Brno, 61242 Brno, Czech Republic; cizekp@vfu.cz

**Keywords:** *Tapirus terrestris*, adaptation to feeding, tongue, Perissodactyla, lingual glands

## Abstract

**Simple Summary:**

This is a detailed study of the surface morphology of the tongue and the lingual glands of the lowland tapir (*Tapirus terrestris*), which expands the understanding of the adaptation of this species to habitats. The histological and ultrastructural analysis of the lingual papillae and lingual glands revealed the presence of two types of mechanical papillae, namely the filiform and conical papillae, while papillae with taste buds (including the fungiform papillae, vallate papillae, and foliate papillae) formed the second, less numerous group. The filiform papillae differed from those of Equidae or Rhinocerotidae. The presence of nine vallate papillae, localized in groups of two surrounded by a ring, or individually, was unique for the examined female tapir. In addition, the vallate papillae contained irregular pseudopapillae on their surface. The foliate papillae contained several sulci between each folia. The presence of sparse taste buds in the side wall of the vallate papillae and foliate papillae is unique for the tapir. Compared to other Perissodactyla, the number of taste buds in the tapir is limited, although the features of its tongue surface make it possible to distinguish this species from representatives of Equidae or Rhinocerotidae.

**Abstract:**

Although the anatomy of the gastrointestinal tract has been characterized in the lowland tapir (*Tapirus terrestris*), the exact anatomy of its tongue has not been studied. Samples of the lingual papillae and lingual glands were collected from the tongue of an adult female lowland tapir. The microscopic analysis of the structure of the lingual papillae and the histochemical analysis of the secretion of the lingual glands were analyzed. The tongue of the tapir is divided into the apex, body with a distinct lingual prominence, and the root. Its ventral surface is smooth. The most numerous of the mechanical papillae were the filiform papillae, while numerous conical papillae with a sharp apex or more rounded papillae were present in the root of the tongue. There were also nine vallate papillae and pair of foliate papillae. The foliate papillae contained several folds parted by 12–14 grooves. The mucous secretion produced by the lingual glands was more obvious than the serous secretion. The features of the dorsal surface of the tongue as well as the shape and number of the lingual papillae on the surface of the tongue of the examined female tapir differ compared to Equidae or Rhinocerotidae, the other two representatives of Perissodactyla. However, further study is necessary for the synapomorpy of the tapir’s tongue.

## 1. Introduction

The lowland tapir (*Tapirus terrestris*) is the most widespread member of the *Tapiridae* (Perissodactyla) family and inhabits the subtropical and tropical zones of South America (northern Argentina, Brazil, Bolivia, Peru, Ecuador, Venezuela, Guyana, Suriname, French Guiana, and Colombia) [1,2]. The lowland tapir is considered a vulnerable species according to the IUCN Red List of Threatened Species [3]. Population declines are caused by habitat eradication, illegal hunting, competition with livestock, deforestation, and other forms of habitat change [1].

The lowland tapir is a herbivore, known to consume a wide variety of plants, but its diet is not fully understood [4]. Tapirs are adapted to habitats, switching their diet from frugivory to herbivory when fruits are scarce [5]. They browse selectively on plant parts, bite off mainly the terminal and the youngest parts because these are more nutritive and less fibrous [6,7].

The tapir diet is highly diverse, including seasonal and locally available plants [8]. The diversity in the consumed plant species and the degree of fruits in the diet are suspected to vary regionally and seasonally, most probably depending on resource availability. Up to 112 plant species were found to be a part of the lowland tapir’s diet [9]. One third of the plants were tree species, another third were shrub species, one quarter were herbs and the rest were lianas [9]. *Melastomataceae*, *Sapotaceae*, *Rubiaceae*, *Araceae*, *Moraceae*, and *Fabaceae* were reported as the most common consumed plants [9,10,11]. According to Chalukian et al. [5], lowland tapirs consumed leaves of several species of plants, stems, fruits, vegetative parts of plants, and flowers. The most frequently eaten fruits were large juicy and fragrant fruits of *Spondias mombin*, *Helicostylis tomentosa*, *Ficus* spp., and *Bagassa guianensis* [9]. Fruit consumption was precisely studied by Bizerril et al. [12] and O’Farrill et al. [13].

The tapir possesses a true proboscis similarly to elephants, showing an evolutionary convergence between the order Perissodactyla and the Proboscidea order [14,15]. Tapirs use their proboscis, at least in part, to grasp food. The grasping action appears to be rudimentary, mainly because its anterior extension beyond the food-grasping incisors is minimal. The labial surface of the *proboscis* pushes food towards the mouth. The narial structures indirectly grasp food [16]. The jaws and the dentition of the tapirs are reduced, despite the recession of the bony nares of the nasal skull and the shortening of the nasal bones [15]. Tapirs have a modified ‘nose and upper lip’ but not a modified jaw, whereas both of these structures are modified in elephants [16].

Although the anatomy of the tapir’s gastrointestinal tract was precisely described [17], the detailed lingual morphology was still unknown. As an odd-toed ungulate (Perissodactyla), the tapir is relative to families *Equidae* and *Rhinocerotidae*. The lingual morphology has already been described in representatives of these families. Filiform papillae seem to be similar in their appearance (hair-like or needle-like) in all described odd-toed ungulates [18,19,20,21,22,23]. Conical papillae were reported in the donkey [18] and the horse [19]. Vallate papillae were described in all species of the *Equidae* and *Rhinocerotidae* families but their number varied from two in the zebra [20] and in the horse [19,23], 2–5 in the donkey [18,22], approximately 60 [21] or 43 [24] in the black rhinoceros (*Diceros bicornis*), 23–29 in the white rhinoceros (*Ceratotherium simum*) [24], and 21–23 in the Indian rhinoceros (*Rhinoceros unicornis*) [24]. Foliate papillae were described in the donkey (*Equus asinus*) [18,22] and in the horse (*Equus caballus*) [19,23]. No foliate papillae were reported in the Chapman‘s zebra (*Equus quagga chapmani*) [20], in the black rhinoceros [21,24], or in the white rhinoceros and the Indian rhinoceros [24]. Round fungiform papillae were reported in all the mentioned odd-toed ungulates [18,19,20,21,22,23,24].

The last family belonging to the Perissodactyla—*Tapiridae*—includes only one genus—*Tapirus*.

The four extant species of tapirs as *Tapirus bairdii*, *Tapirus indicus, Tapirus pinchaque,* and *Tapirus terrestris* are known [3]. So far, no microscopical study of the tongue has been performed in the tapir, while macroscopic examination has been performed by Sonntag, 1922 [25]. The aim of this study is to provide microscopic information on the tongue of the tapir with relation to its phylogenesis, feeding habits, as well as its very specific morphology. The composition of the lingual glands was also investigated.

## 2. Materials and Methods

### 2.1. Collection of the Tongue

The tongue was collected in 2019 from an adult female lowland tapir (*Tapirus terrestris*) at the Wroclaw Zoo (Wrocław, Poland). The animal was 25 years, 3 months, and 27 days old. The District Veterinary Officer permitted sample collection (UT-45/5/16; 45/6/16; UT-45/8/16). Pursuant to the law in the European Union, no consent of the local Ethics Committee for Animal Experiments is required for post-mortem sampling.

The tongue was collected immediately after the animal’s death due to natural causes. Post mortem examination of animal showed that the leading cause of death was the cardiac tamponade. Additionally, the weak condition of animal was observed because of the dental problems. The length, width, and thickness of the tongue was measured with an electronic caliper an accuracy of 0.1 mm. Subsequently, the collected sample was photographed (Canon EOS 300, Canon, New York, NY, USA) and a macroscopic analysis of the surface of the tongue was performed (Zeiss Stemi 2000-C stereoscopic microscope (Carl Zeiss, Jena, Germany)).

### 2.2. Histological and Histochemical Analysis

Samples of the lingual papillae and lingual glands were collected from the apex, body, lingual prominence, and root of the tongue for histological and histochemical studies. All samples were placed in 10% buffered neutral formalin (Chempur, Piekary Śląskie, Poland). After at least 72 h the specimens were washed in running water (24 h) and dehydrated in a series of alcohol dilutions (50%, 75%, 96%, and 100%), then cleared in xylen. In the next step the samples were impregnated with paraffin and cut with using of microtome Slide 2003 (Pfm A.g, Cologne, Germany)) into 4 µm sections. The samples then underwent routine staining procedures using Masson-Goldner trichrome and Azan trichrome staining. Alcian blue pH 1.0 (AB pH 1.0), alcian blue pH 2.5 (AB pH 2.5), PAS-AB pH 2.5, periodic-acid Schiff (PAS), and Hale’s dialysed iron (HDI) staining methods were used to determine the type of lingual gland secretions [26,27]. The results of the histochemical stains were assessed using the methodology described by Spicer and Henson, 1969 [28], where (−) indicated a negative reaction, (+) a weak positive reaction, (++) a mild positive reaction, and (+++) a strong positive reaction. Microscopic photos were taken using a Zeiss Axio Scope A1 light microscope (Carl Zeiss, Jena, Germany).

### 2.3. Ultrastructural Analysis—Scanning Electron Microscopy

The samples of the lingual papillae were collected for ultrastructural analysis from the apex, body, lingual prominence, and root of the tongue and placed in 2% glutaraldehyde diluted in 0.1 M PBS at a pH 7.4. The samples were dehydrated in a graded ethanol series (30%, 50%, 70%, 80%, 90%, 96%, and 100%, 3 × 10 min for each concentration), transferred to absolute acetone [29] and then dried using Bal-tec CPD 128 030 Critical Point Dryer (Bal-Tec, Reading, UK). They were then coated with gold (Balzers SCD 040 by current 30 mA for 4 min) and visualized using a Tescan VEGA TS 5136 XM microscope (Tescan, s.r.o., Brno, Czech Republic).

The obtained results were described using the Nomina Anatomica Veterinaria [30] and Nomina Histologica Veterinaria [31].

## 3. Results

### 3.1. Macroscopic Findings of the Surface of the Tongue

**Figure 1:**
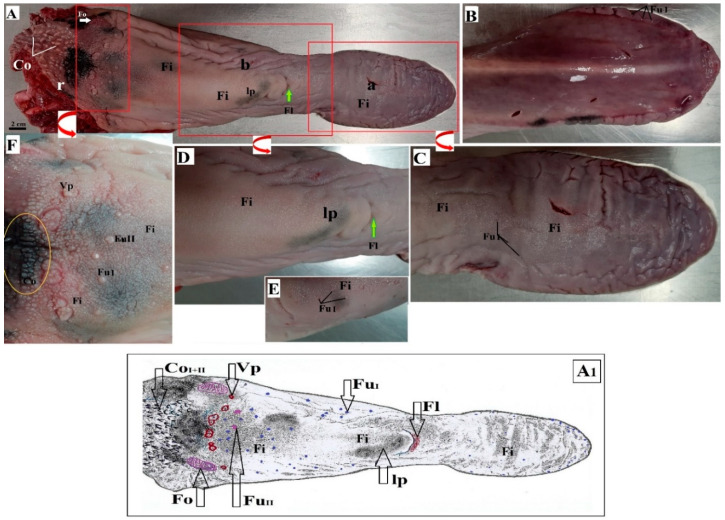
Photographs and diagram of the tongue of the female of *Tapirus terrestris*. (**A**) Dorsal surface of the tongue. White arrow—foliate papilla. Green arrow—fossa lingualis. Bar = 2 cm. **A_1_**. Diagram of the dorsal surface of the tongue; (**B**) smooth ventral surface of the apex of the tongue; (**C**) dorsal surface of the apex of the tongue with lingual papillae; (**D**) lingual prominence—magnification; (**E**) magnification of the fungiform papillae on the dorsal surface of the body of the tongue; (**F**) magnification of the root of the tongue with the vallate papillae, fungiform papillae, conical papillae, and filiform papillae. Yellow circle—area with the darkest pigmentation of the dorsal surface of the tongue. Abbreviations: a—apex of the tongue; b—body of the tongue; Co—conical papilla; Co_I_—type I of conical papilla; Co_II_—type II of conical papilla; Fi—filiform papilla; Fl—fossa lingualis; Fu I—type I of fungiform papilla; Fu II—type II of fungiform papilla; lp—lingual prominence; r—root of the tongue; Vp—vallate papilla.

The tongue of the lowland tapir consisted of three parts, namely the apex, body, and root. The lingual prominence was well marked within the body of the tongue. The tongue was 35 cm long. It was almost 8 cm wide at the apex, 6 cm wide at the body, and 11 cm wide at the root. The thickness of the apex was 2.5 cm, 6 cm at the body, and 8.5 cm at the root, respectively. There was no dark pigmentation on the dorsal surface of the tongue within the apex, there was scarce pigmentation on the lingual prominence and most pigmentation on the surface of the root of the tongue and on some lingual papillae (Figure 1A). Moreover, there was no visible median groove on the dorsal surface of the tongue. The apex of the tongue was rounded (Figure 1A,B). The body of the tongue was narrowed directly before the lingual prominence, while the presence of the fossa lingualis was noted rostral to the lingual prominence (Figure 1A,D). The ventral surface of the tongue was smooth and did not contain papillae (Figure 1B). The filiform papillae were the most numerous mechanical papillae and covered the dorsal surface of the apex, body, and lingual prominence as well as part of the root of the tongue. The less numerous conical papillae were located around the vallate papillae (Figure 1A,F), while the largest conical papillae were seen caudally to the vallate papillae (Figure 1F).

The gustatory papillae consisted of vallate papillae, fungiform papillae, and foliate papillae (Figure 1A,C,D,F). Two types of fungiform papillae were noted. Type I round fungiform papillae scarcely located on the apex, body, and partially on the root of the tongue, while type II bean-shaped fungiform papillae were larger than type I papillae (few) (Figure 1A,E,F). The largest fungiform papillae (16, both type I and type II) were present directly rostrally to the vallate papillae. Moreover, round fungiform papillae were more numerous on the lateral surface of the apex and body of the tongue (they were large close to the ventral surface of the apex) (Figure 1B). Nine vallate papillae located in a semicircle between the body and root of the tongue were also distinguished (Figure 1A). The arrangement of these papillae was irregular: six were dispersed in pairs, while three were single. All of the vallate papillae were surrounded by filiform papillae, so that no distinct ridge was seen surrounding the papillae (Figure 1F). The surface of each vallate papilla was covered by several pseudopapillae. A pair of large foliate papillae was found directly behind the vallate papillae on the caudolateral surface of the root of the tongue (Figure 1A). The foliate papillae on the left and right comprised 12–14 folia divided by sulci. The majority of the sulci was arranged parallel, although some were half as long as the other sulci, giving a letter Y shape to some folia (Figure 1A).

### 3.2. Lingual Papillae—Histological Analysis and SEM Observations

#### 3.2.1. Filiform Papillae

**Figure 2:**
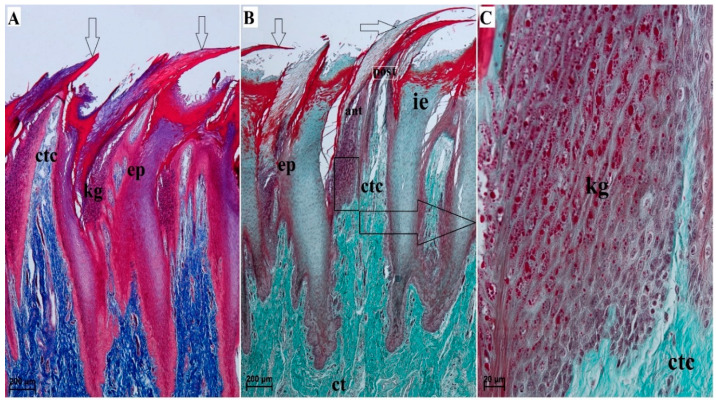
Histomicrograph of the filiform papillae of the tongue of the female of *Tapirus terrestris*. (**A**) Longitudinal section of the filiform papillae. Azan trichrome staining. Bar = 200 µm; (**B**) magnification of filiform papillae with keratinized stratified squamous epithelium and connective tissue core—well defined superficial layer as additional processes. Masson–Goldner trichrome staining. Bar = 200 µm. (**C**) Magnification of the granular layer of keratinized stratified squamous epithelium with well visible numerous keratohyaline granules—with different shape and size of these granules. Masson–Goldner trichrome staining. Bar = 20 µm. Abbreviations: ant—anterior cell column; ct—connective tissue; ctc- connective tissue core; ep—stratified squamous epithelium; ie—interpapillary epithelium; kg—keratohyaline granules; white arrows—processes of the filiform papillae; post—posterior cell column.

The filiform papillae were covered by a stratified squamous epithelium with varying degrees of keratinization. The filiform papillae varied between the apex, body, and root of the tongue. A histologic analysis revealed the presence of an anterior column of cells and a posterior column of cells forming the filiform papillae (Figure 2A,B). The degree of epithelial keratinization varied. There were also numerous keratohyaline granules of various shape in the *stratum granulosum* (Figure 2C). An interpapillary epithelium was present among the filiform papillae (Figure 2A,B).

**Figure 3:**
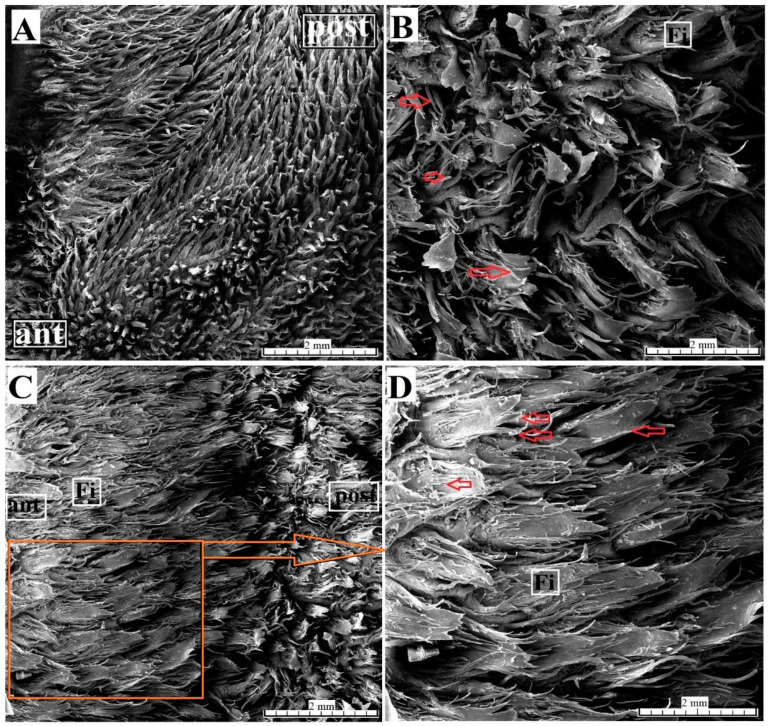
SEM analysis of the filiform papillae of the apex of the tongue and lingual prominence of the female of *Tapirus terrestris*. (**A**) Numerous filiform papillae from apex of the tongue. Bar = 2 mm; (**B**) magnification of the filiform papillae from lingual prominence of the tongue. Note several secondary processes on each filiform papilla—red arrows. (**C**) Numerous filiform papillae composed of main papilla and secondary processes on lingual prominence. Bar = 2 mm. (**D**) Magnification of the filiform papillae from lingual prominence. Note several secondary processes on each filiform papilla—red arrows. Bar = 2 mm. Abbreviations: ant—rostral; Fi—filiform papilla; post—caudal.

The SEM study revealed that the filiform papillae on the apex of the tongue consisted of a main part and small additional processes (Figure 3A). The filiform papillae on the lingual prominence consisted of a main leaf and several long secondary processes (Figure 3B). In addition, some of the main leaves of the filiform papillae on the lingual prominence had a divided apical part (Figure 3C,D) but also differed from the filiform papillae located close to the vallate papillae. The filiform papillae on the lateral surface of the body of the tongue were elongated and contained no secondary papillae.

#### 3.2.2. Conical Papillae

**Figure 4:**
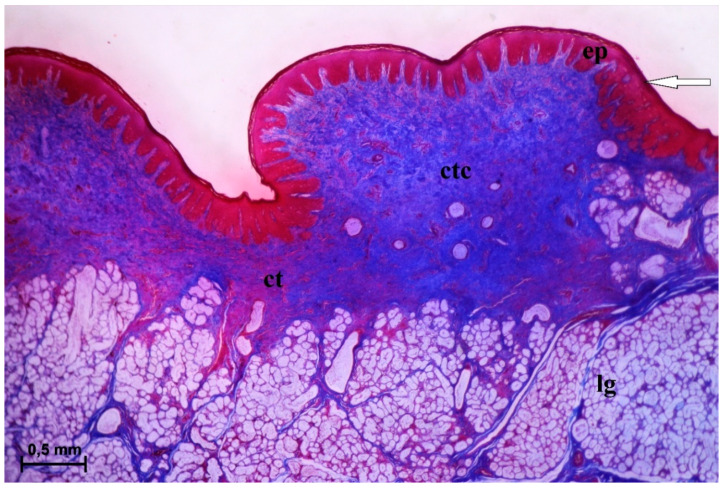
Histomicrograph of the conical papilla of the tongue of the female of *Tapirus terrestris*. Longitudinal section of the conical papilla from root of the tongue with well visible stratum corneum of the epithelium—white arrow. Note the mixed deep lingual glands of the root of tongue. Azan trichrome staining. Bar = 0.5 mm. Abbreviations: ct—connective tissue; ctc—connective tissue core; ep—keratinized stratified squamous epithelium; lg—lingual glands.

**Figure 5:**
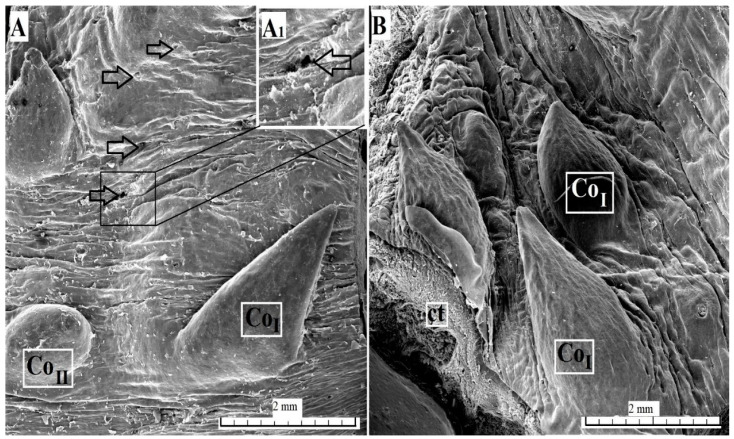
SEM analysis of the conical papillae of the tongue of the female of *Tapirus terrestris*. (**A**) Conical papillae with different size on the surface of the root of the tongue with numerous openings of the lingual glands—white arrows. Bar = 2 mm. **A_1_** Higher magnification of the opening of lingual glands; (**B**) four conical papillae. Bar = 2 mm. Abbreviations: Co_I_—conical papilla type I; Co_II_—conical papilla type II; ctc—connective tissue core.

The conical papillae present on the surface of the root of the tongue were covered with keratinized stratified squamous epithelium (Figure 4). A layer of the *stratum corneum* was obvious. The connective tissue core contained numerous blood vessels, connective tissue penetrating the epithelium and forming numerous elongated papillae (Figure 4). A part of the conical papillae had a pointed apex, type I (Figure 1A), while the remaining papillae had a rounded apex, type II (Figure 1A and Figure 4). The SEM analysis revealed that the type I conical papillae were triangular, while the type II conical papillae were more lens-shaped (Figure 5). The surface of the conical papillae was smooth without secondary papillae (Figure 5).

#### 3.2.3. Fungiform Papillae

**Figure 6:**
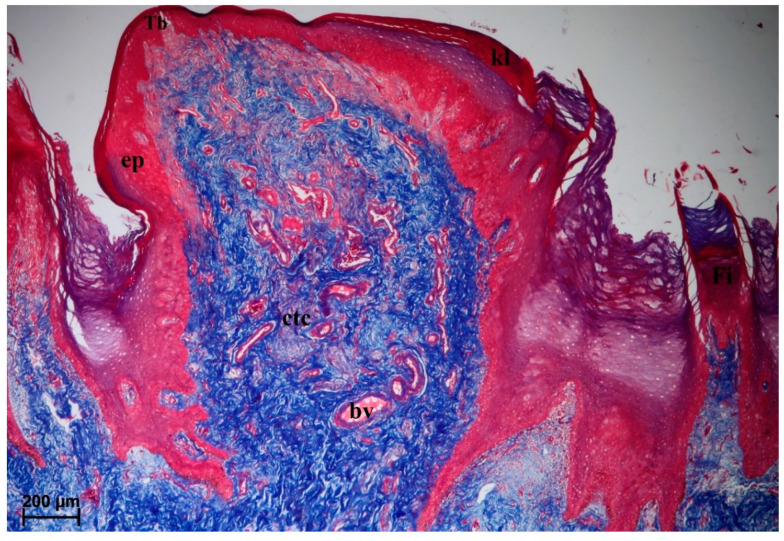
Histomicrograph of the fungiform papilla of the tongue of the female of *Tapirus terrestris*. Longitudinal section of the round fungiform papilla from body of the tongue with a well visible keratinized superficial layer of the epithelium—note the presence of taste bud within the epithelium. Azan trichrome staining. Bar = 200 µm. Abbreviations: bv—blood vessels; ctc—connective tissue core; ep—keratinized stratified squamous epithelium; Fi—filiform papilla; kl—keratinized cells; Tb—taste bud.

**Figure 7:**
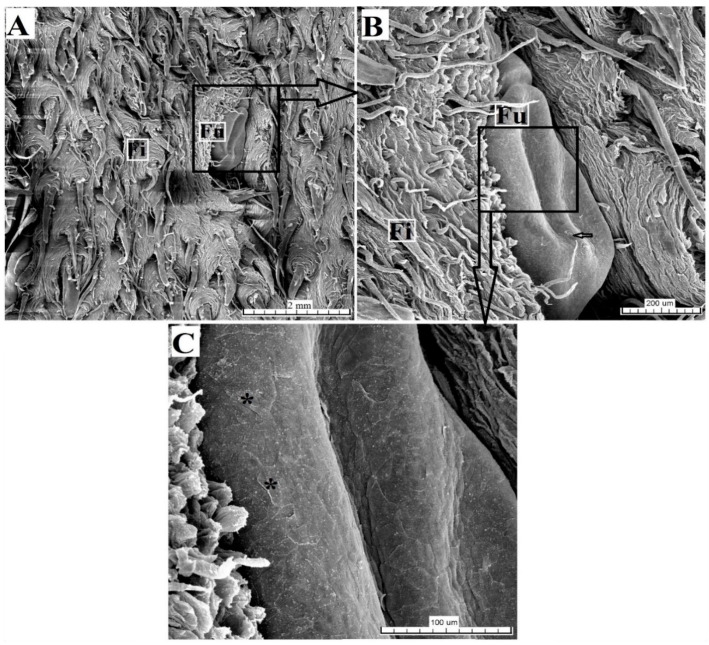
SEM analysis of the fungiform papillae and filiform papillae of the tongue of the female of *Tapirus terrestris*. (**A**) Three round fungiform papillae from lateral surface of the body of tongue surrounded by numerous filiform papillae. Bar = 2 mm. (**B**) Magnification of fungiform papillae with opening of taste pore (arrow). Bar = 2 µm. (**C**) Magnification of the dorsal surface of fungiform papilla with well visible exfoliating cells (asterisks). Abbreviations: ant—rostral; Fi—filiform papilla; Fu—fungiform papilla; post—caudal.

The surface of the fungiform papillae was covered with a keratinized stratified squamous epithelium with a visible *stratum corneum* (Figure 6). One taste bud was visible on the dorsal surface of the fungiform papillae (Figure 6). The connective tissue core contained numerous blood vessels (Figure 6). The SEM analysis revealed that the edges of the fungiform papillae were smooth (Figure 7A–C). The openings of the taste pores were sparsely deployed on the surface of fungiform papilla (Figure 7B,C). Moreover, some of the fungiform papillae on the lateral surface of the body of the tongue were located in depressions and surrounded by a ridge.

#### 3.2.4. Vallate Papillae

**Figure 8:**
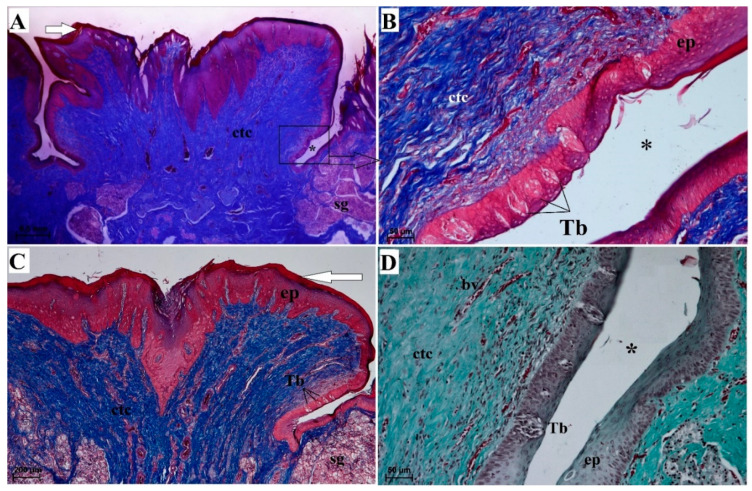
Histomicrograph of the vallate papilla of the tongue of the female of *Tapirus terrestris*. (**A**) Longitudinal section of the vallate papilla of the tongue with a well visible keratinized superficial layer of the epithelium (white arrow)—note the presence of irregular surface of this papilla. Azan trichrome staining. Bar = 0.5 cm. (**B**) Magnification of the lateral wall of the vallate papilla at their base—note the presence of several taste buds within the epithelium of this wall. Azan trichrome staining. Bar = 50 µm. (**C**) Magnification of the epithelium and connective tissue core of vallate papilla—note the presence of serous glands beneath of the papilla and keratinized superficial layer of the epithelium (white arrow). Bar = 200 µm. (**D**) Magnification of the lateral wall of the vallate papilla—note the presence of sparse taste buds within the epithelium of this wall. Masson–Goldner trichrome staining. Bar = 50 µm. Abbreviations: bv—blood vessels; ctc—connective tissue core; ep—keratinized stratified epithelium; sg—serous glands; Tb—taste bud; * (asterisk)—groove of the papilla.

**Figure 9:**
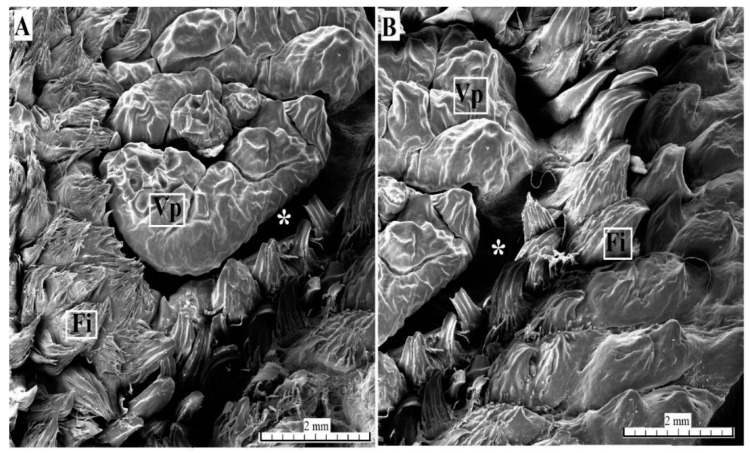
SEM analysis of the vallate papillae of the tongue of the female of *Tapirus terrestris*. (**A**) Vallate papilla with irregular surface (several pseudopapillae)—note the absence of smooth annulary pad around of this papilla. Bar = 2 mm. (**B**) Magnification of the vallate papilla surface—note the presence of papillary groove around of the papilla and numerous mechanical papillae which surrounded of vallate papilla. Bar = 2 mm. Abbreviations: Fi—filiform papilla; Vp—vallate papilla; * (asterisk)—groove of the papilla.

The surface of the vallate papillae was covered by a keratinized stratified squamous epithelium with a visible thin *stratum corneum* (Figure 8A,C). There were numerous blood vessels in the connective tissue core (Figure 8A,C). The lateral wall of the vallum of the vallate papillae contained sparse taste buds (Figure 8A–D). Serous glands (von Ebner’s glands) were present at the base of the vallate papilla (Figure 8A–C). The SEM analysis revealed an irregular surface of the vallate papillae, covered by numerous pseudopapillae of various sizes (Figure 9A,B). Filiform papillae and conical papillae were noted around the vallate papillae and contained a main part as well as several secondary processes. Some of those filiform papillae were smaller and contained no additional processes, while others contained several secondary processes (Figure 9A). Hence, there was no typical smooth annular pad, but instead rows around the papilla (Figure 9B) or around pairs of papillae, if they lay adjacent (Figure 1F).

#### 3.2.5. Foliate Papillae

**Figure 10:**
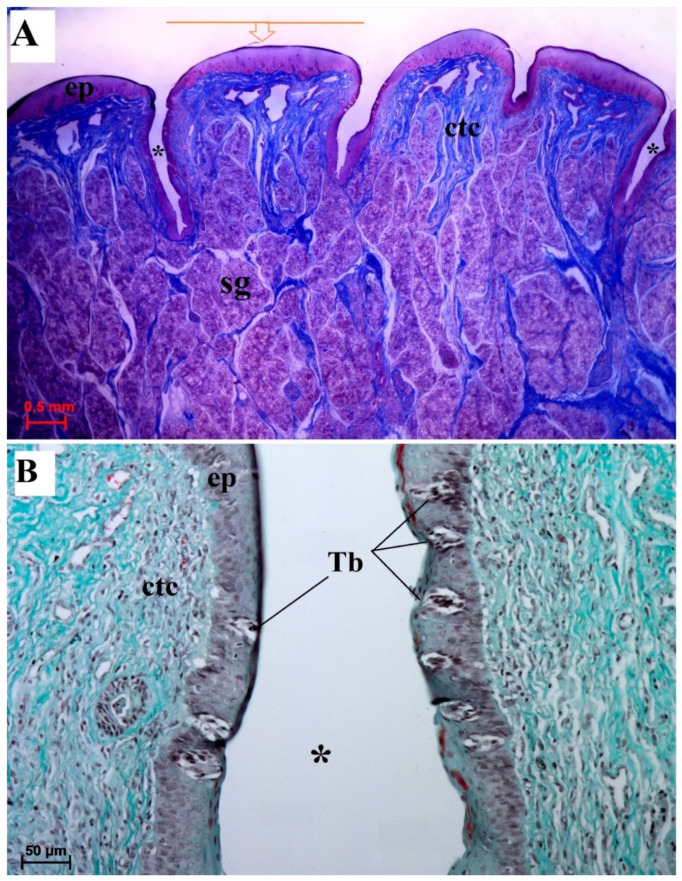
Histomicrograph of the foliate papilla of the tongue of the female of *Tapirus terrestris*. (**A**) Longitudinal section of the foliate papilla of the tongue—note the presence of the several foliae (single folia signed with orange arrow) and grooves between of each folia, and presence of serous glands beneath of the base of papilla. Azan trichrome staining. Bar = 0.5 cm. (**B**) Magnification of the lateral wall of the folia of the foliate papilla at their base—note the presence of several taste buds within the epithelium of this wall. Masson–Goldner trichrome staining. Bar = 50 µm. Abbreviations: ctc—connective tissue core; ep—stratified squamous epithelium; sg—serous glands; Tb—taste bud; * (asterisk)—groove of the papilla.

The foliate papillae were covered by a keratinized stratified squamous epithelium with a very thin *stratum corneum* (Figure 10A). The folia of the foliate papillae were separated by sulci of various depths (Figure 10A). Single barrel-shaped taste buds were present in the lateral wall of each folium (Figure 10B).

**Figure 11:**
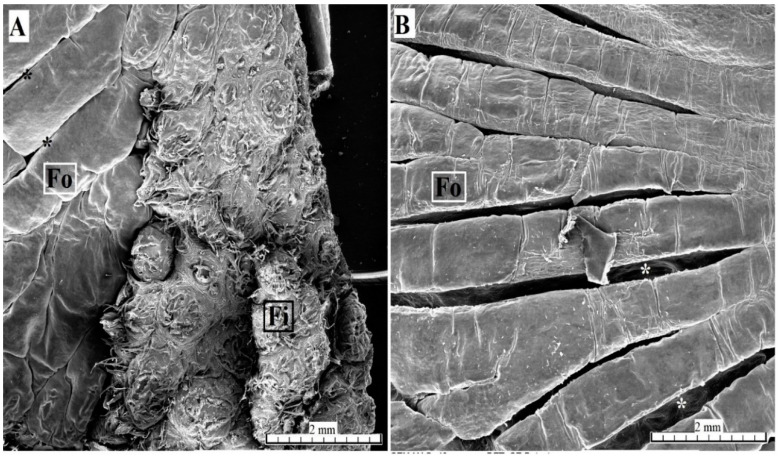
SEM analysis of the foliate papillae of the tongue of the female of *Tapirus terrestris*. (**A**) Foliate papilla with several folia and several longitudinal sulcuses (asterisks). Bar = 2 mm. (**B**) Magnification of the several sulcuses and folia of the foliate papilla—sulcuses (asterisks). Bar = 2 mm. Abbreviations: Fi—filiform papilla; Fo—foliate papilla; * (asterisk)—groove of the papilla (sulcuses).

Serous glands (von Ebner’s glands) were clearly visible at the base of the foliate papillae. The SEM analysis found that the surface of individual folia was smooth (Figure 11).

#### 3.2.6. Lingual Glands—Histochemical Analysis

**Figure 12:**
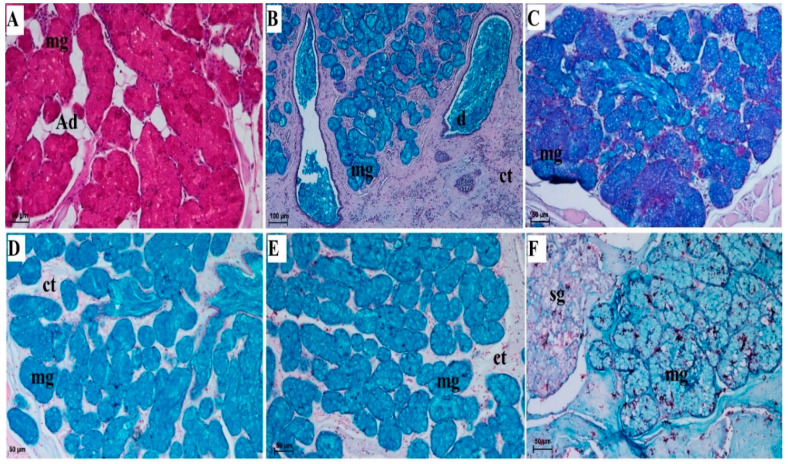
Histochemical analysis of the mucoserous lingual glands of root of the tongue (see lingual zone on Figure 1A—r) of the female of *Tapirus terrestris*. (**A**) Strong positive reaction +++ within the mucous cells of the acini of mucoserous lingual glands. PAS staining. Bar = 50 µm. (**B**) Strong positive reaction +++ within the mucous cells of acini of mucoserous lingual glands. Well visible two main ducts with a mucous inside. PAS-AB pH 2.5 staining—blue color of mucous cells. Bar = 100 µm. (**C**) Strong positive reaction +++ within the mucous cells of the acini of mucoserous lingual glands—magnification. PAS-AB pH 2.5 staining. Bar = 50 µm. (**D**) Strong positive reaction +++ in the mucous cells of the acini of the mucoserous lingual glands—blue color. AB pH 1.0 staining. Bar = 50 µm. (**E**) Strong positive reaction within the acinar cells of mucoserous lingual glands +++ dark blue color. AB pH 2.5 staining. Bar = 50 µm. (**F**) Weakly positive reaction + within mucous cells and negative reaction within serous cells of mucoserous lingual glands. HDI staining. Bar = 50 µm. Abbreviations: Ad—adipose cell; bv—blood vessel; ct—connective tissue; d—duct; mg—mixed glands; sg—serous glands.

The lingual glands comprised accessory serous glands of the gustatory papillae (vallate papillae and foliate papillae) (Figure 8A and Figure 10A) and dominant mixed glands (Figure 4) of the root of tongue (with predominantly mucous units) (Table 1). The serous glands were localized within the connective tissue stroma under the gustatory papillae and produced a secretion that rinsed the sulci around the vallate papillae or those within the foliate papillae. Mixed glands were much more complex in their structure, as they contained both mucous acini and fewer serous acini. The secretions of both units ran to the main ducts and through their openings onto the surface of the root of the tongue. Numerous adipose cells were present between lingual gland parenchyma (Figure 12A). PAS staining showed a strong positive reaction within the mucous cells of the acini (Figure 12A) indicating the presence of neutral glycoconjugates (magenta) in the secretion of those cells. PAS-AB pH2.5 staining showed a strong positive reaction (blue) within the mucous cells of the acini, and a weakly positive reaction within the duct cells (Figure 12B,C). Additionally, PAS-AB pH2.5 stains serous cells of the acini in bright magenta color (Figure 12C). AB pH 2.5/PAS sequence display cellular combinations of both acidic and neutral glycoconjugates (violet). AB pH 1.0 showed a strong positive reaction within the mucous cells of the acini what displays sulphated glycoconjugates (blue) (Figure 12D). AB pH 2.5 (Figure 12E) stains sulphate esters and carboxyl groups in glycoconjugates and in the present study this staining showed a strong positive reaction in the mucous cells of the acini of the lingual glands, while negative reaction in the serous cells of the acini. The HDI staining (for sulfated acid mucopolysaccharydes or carboxylated acid mucopolysaccharydes) showed a weakly positive reaction within the mucous cells (Figure 12F), while negative reaction within serous cells of acini (Figure 12F).

## 4. Discussion

### 4.1. The Tapiridae Tongue Versus the Tongue of Equidae and Rhinocerotidae—Similarities and Differences

All three families, the Tapiridae, Equidae, and Rhinocerotidae, belong to the Perissodactyla order. However, their diet and living environment differ greatly. Although their functional anatomy is similar, the structural features of their individual organs varies. A detailed analysis of the morphometric features allows better understanding of the functional anatomy of the digestive tract. A properly adapted stomach and intestine enable absorption of nutrients by a particular species, although the preprocessing of food by the tongue and teeth, palate, and cheeks also play an important role in this process.

The tongue of the captive lowland tapir differed macroscopically compared to the Equidae [19,21,22,23] or Rhinocerotidae [21,24]. The lingual tonsil was well visible on both sides of the caudal surface of the root of the tongue in *Equus asinus* [18]. This tonsil was not seen in the examined lowland tapir. The apex of the tongue had a triangle shape in the Rhincerotidae [21,24], while it was more rounded in the lowland tapir and comparable to that of the Equidae [18,20,22,32]. The lingual prominence was visible in the lowland tapir, as in *Equus asinus* [18,22], *Equus quagga chapmani* [20], and various representatives of the Rhinocerotidae family [24]. The lingual prominence differed in shape within particular species of the Rhinocerotidae [24] while it was clearly visible in the lowland tapir. The development of this part of the body of the tongue is related to its function of grinding plants, although this lingual prominence lacked conical mechanical papillae. The median groove was not present in the tongue of the lowland tapir, which was present on the apex of the tongue in *Equus asinus* [18] and *Equus quagga chapmani* [20].

The number and distribution pattern of individual lingual papillae differed between species of the three families in the Perissodactyla order. The main difference was in the number and distribution of the gustatory papillae on the surface of the tongue of the lowland tapir. The filiform papillae were the most numerous mechanical papillae in the lowland tapir, similarly to Equidae [18,20] or Rhinocerotidae [21].

The filiform papillae present on the apex of the tongue in the lowland tapir contained fewer secondary processes. In contrast to the filiform papillae in the lowland tapir, there were two types of filiform papillae in the *Equus asinus* [22] and no conical papillae. Similarly, there were two types of conical papillae in the lowland tapir. Needle-like and hair-like conical papillae were found in the *Equus quagga chapmani* [20]. The shape of the filiform papillae in the lowland tapir was comparable to that in the black rhinoceros, where the main papillae contained several additional processes, whereby those on the body of the tongue were larger than those on the apex [21]. The filiform papillae were studied using SEM analysis in only one other Rhinocerotidae species, namely in the black rhinoceros [21]. Hence, it is difficult to compare the obtained results of the lowland tapir with other species of this family. In the case of horses, long and slender filiform papillae were found on the rostral surface of the tongue and two types of filiform papillae were observed on the lingual prominence [23]. Similarly, Can et al. confirmed the presence of varying types of filiform papillae on the apex and lingual prominence in horses [19] which is comparable to the results found in the lowland tapir. The conical papillae in the lowland tapir were of two types which differed in size and shape of their apex. Similar conical papillae were present in the Rhincerotidae [24], whereby Cave (1977) also observed single lenticular papillae. No conical papillae were present in the Equidae [18,19,20].

Interestingly, two types of fungiform papillae were identified, which can be compared to the presence of the second type of “lobulated” fungiform papillae in the *Equus asinus* [18]. Moreover, the remaining round fungiform papillae (type I papillae) were comparable to those occurring in certain Rhinocerotidae, such as the Indian rhinoceros [24], the white rhinoceros [24] and black rhinoceros [21,24], or in the *Equus quagga chapmani* [20] and *Equus asinus* [22]. However, the *Equus asinus* lacked taste buds in the apical part of the fungiform papillae [18], while single taste buds were observed in the lowland tapir. According to Can et al. (2016), a part of the equine fungiform papillae was round, while some were lobulated [19] without taste buds. However, Chamorro et al. [33] and Pfeiffer et al. [32] obtained different results and noted the presence of taste buds and their taste pores on fungiform papillae, similarly to what we observed in the lowland tapir. The results of different studies into fungiform papillae in various Equidae species revealed that they have a mechanical function if taste buds are absent [18,33]. The presence of the taste buds confirms their gustatory function.

The arrangement and shape of the eight vallate papillae in the lowland tapir differed from other representatives of the Perissodactyla order. Contrary to the lowland tapir, representatives of Equidae, such as the *Equus asinus*, contained from 2–5 vallate papillae [22] of various shapes and a flat surface of 2–3 vallate papillae [18]. There were more taste buds in the lateral wall of the vallate papilla in *Equus asinus* [18,22] compared to the lowland tapir, where fewer taste buds were arranged at the basis of the vallum of the papillae. There was also a significant difference in the number of vallate papillae in the horse compared to the lowland tapir, as the former had two round vallate papillae [23], while the latter had six connected vallate papillae that extended in pairs and had an irregular shape. The *Diceros bicornis* had, in contrast, 60 [21] or 43 [24] vallate papillae, which were round, lay adjacent, and were irregularly dispersed along both sides of the root of the tongue. The macroscopic examination of the white rhinoceros and Indian rhinoceros also revealed the presence of several vallate papillae [24], more than what was recorded in the studied lowland tapir. To date, a histological analysis of the vallate papillae in the Rhinocerotidae has not been performed, hence the presence, number, and structure of the taste buds [21,24] in the lateral wall of the vallate papilla in the Rhinocerotidae cannot be compared with that of the lowland tapir. One similarity between the lowland tapir and Rhinocerotidae was the presence of adjacent vallate papillae [21,24].

A pair of foliate papillae was present in the lowland tapir similarly to horse [19,23,32,33] or donkey [18,22]. The foliate papillae in the *Equus asinus* contained fewer mucosal folds than the tapir, namely 7–8 [22] or 8–10 [18], which also contained numerous taste buds [18]. Unlike in the lowland tapir, there were no foliate papillae in the black rhinoceros [21]. In the remaining studied Rhinocerotidae species, no foliate papillae were found [24]. In the horse, many taste pores of taste buds were observed in the lateral wall of the mucosal folds of the foliate papillae [19] which was similar to the observation made in the lowland tapir. Similarly, in the donkey, numerous taste buds were found in the mucosal folds of the foliate papillae [18,22].

The glands of the tongue in the lowland tapir comprised serous von Ebner’s glands, accessory vallate papillae, and foliate papillae, while units of mixed glands of the root of the tongue were better developed. The secretion of these glands opened on the surface of the root of the tongue between numerous conical papillae. Histochemically, sulfated mucins and carboxylated sialomucins predominated in the mixed glands. Due to no histochemical studies of the tongue in the Rhinocerotidae, the histochemical analysis cannot be compared between animals from this family. On the other hand, the content of lectins in the secretory cells and duct cells of von Ebner’s glands has been studied in horses [34].

### 4.2. The Tongue of the Tapirus Terrestris in Relation to a Diverse Diet in Chosen Mammals

The diet of the lowland tapir focuses on certain plants but occasionally also includes fruits [2,4,5]. Therefore, in this section, features of the tongue of the lowland tapir were compared with those of other plant-eating species. From the Equidae, Rhinocerotidae, and Tapiridae families in the Perissodactyla order, the Tapiridae have the most diverse diet [2].

#### 4.2.1. Folivorous and Graminivorous Diet

Many representatives of Artiodactyla [35,36,37,38,39,40,41,42,43,44,45,46,47,48] have a lingual prominence, which was also observed in the lowland tapir. The main difference in this prominence between animals from the two orders is the presence of much larger conical or lenticular papillae in the Artiodactyla [35,38,46] which are absent in the lowland tapir (apart from conical papillae within the root of the tongue). Moreover, the pigmentation on the dorsal surface of the tongue is present to various degrees in the Artiodactyla, while it is distinct in the sitatunga [38]. The most discernable pigmentation in the lowland tapir was on the surface of the root of tongue. Another feature of the Artiodactyla [35,36,37,38,40,41,42,43,44,45,46] is a lack of foliate papillae. These were well developed in the lowland tapir. The lesser mouse deer, a member of the Artiodactyla order, is an exception, as it contains foliate papillae on its tongue [39]. The number and shape of the vallate papillae differs between the lowland tapir and the Artiodactyla, where there are from 1 to 5 vallate papillae on both sides of the caudolateral aspect of the tongue [45] in the alpaca, 26 vallate papillae [42] in the goitered gazelle, and 6–8 on both sides of the caudolateral aspect in the bactrian camel [47]. Histochemical analysis of the lingual glands of lowland tapir showed the similarity especially of von Ebner’s glands secretion to the same glands in sheep [49]. On the other hand, there were differences in secretion nature of the mucoserous glands of the root of tongue between lowland tapir and sheep [49]. However, the mucous units of posterior lingual glands were dominant both in sheep [49] as well as in examined lowland tapir.

In Marsupialia with a folivorous diet, i.e., the bear cuscus [50] and koala [51], there was no lingual prominence, which is another feature distinguishing this infraclass from the Tapiridae family. This is also the case with the Pilosa order i.e., the maned sloth [52], which feeds mainly on liana leaves. The comparison of specific folivorous animals is limited to leaf-eaters. The presence of foliate papillae comparable to the ones in the lowland tapir was only confirmed in the bear cuscus [50], although their size and shape varied slightly. The number of taste buds in the lowland tapir was limited, while there were abundant taste buds in the lateral wall of the foliate papillae in the bear cuscus [50].

#### 4.2.2. Frugivorous Diet

Adaptation to a fruit diet is seen in typically frugivorous animals, such as some members of the Chiroptera order. Interestingly, the filiform papillae are most varied and have been found to exist in 5–6 types, depending on the fruit bat species [53,54,55]. The lesser dog-faced fruit bat [53], large flying fox [54], and Egyptian rousette bat [55] lack foliate papillae, which is contrary to the findings in the lowland tapir, which occasionally eats fruit. However, in the above listed fruit bats, the number of vallate papillae is smaller (only two) compared to the lowland tapir.

## 5. Conclusions

Compared to other Perissodactyla, the number of taste buds in the tapir’s tongue was limited, although the features of its lingual surface make it possible to distinguish this species from representatives of Equidae or Rhinocerotidae. On the other hand, the number of examined samples was reduced thus the similarities and/or differences between female and male of *Tapirrus terrestris* were not detected. The subsequent question is if there are some differences in the lingual surface microstructure of the tongue in accordance with the diet of the captive and wild tapirs? Thus, the further study are necessary for the synapomorpy of the tapir’s tongue.

## Figures and Tables

**Table 1:** Histochemical results of the lingual glands analysis of the female of *Tapirus terrestris.*

	Lingual Glands	Von Ebner’s Glands Beneath the Vallate Papillae and Foliate Papillae	Mucoserous Glands from Root of the Tongue
Staining Method			
PAS		-	+++ cells of mucous acini + cells of duct
PAS-AB pH 2.5		+ cells of serous acini: bright magenta	+++ cells of mucous acini: blue ++ cells of duct: blue + cells of serous acini: bright magenta
AB pH 1.0		-	+++ cells of mucous acini + cells of duct
AB pH 2.5		-	+++ cells of mucous acini + cells of duct
HDI		-	+ cells of mucous acini

Results are expressed by a subjective scale: -, negative reaction; +, weak positive reaction; ++, mild positive reaction; +++, strong positive reaction.
